# Spatial intimacy of binary active-sites for selective sequential hydrogenation-condensation of nitriles into secondary imines

**DOI:** 10.1038/s41467-021-23705-9

**Published:** 2021-06-07

**Authors:** Sai Zhang, Zhaoming Xia, Yong Zou, Mingkai Zhang, Yongquan Qu

**Affiliations:** 1 Key Laboratory of Special Functional and Smart Polymer Materials of Ministry of Industry and Information Technology, School of Chemistry and Chemical Engineering, Northwestern Polytechnical University, Xi’an, China; 2 Center for Applied Chemical Research, Frontier Institute of Science and Technology, Xi’an Jiaotong University, Xi’an, China

**Keywords:** Catalyst synthesis, Catalytic mechanisms, Heterogeneous catalysis

## Abstract

Precisely controlling the spatial intimacy of multiple active sites at sub-nanoscale in heterogeneous catalysts can improve their selectivity and activity. Herein, we realize a highly selective nitrile-to-secondary imine transformation through a cascaded hydrogenation and condensation process by Pt_1_/CoBO_x_ comprising the binary active sites of the single-dispersed Pt and interfacial Lewis acidic B. Atomic Pt sites with large inter-distances (>nanometers) only activate hydrogen for nitrile hydrogenation, but inhibit condensation. Both adjacent B…B on CoBO_x_ and neighbouring Pt…B pairs with close intimacy of ~0.45 nm can satisfy the spatial prerequisites for condensation. Mechanism investigations demonstrate the energetically favorable pathway occurred on adjacent Lewis acidic B sites through the nitrile adsorption (acid-base interaction), hydrogenation via hydrogen spillover from Pt to B sites and sequential condensation. Strong intermolecular tension and steric hindrance of secondary imines on active sites lead to their effective desorption and thereby a high chemoselectivity of secondary imines.

## Abstract (web summary)

Precisely controlling the spatial intimacy of multiple active sites in heterogeneous catalysts can significantly affect the selectivity and activity. Here the authors show a binary active site of single atom Pt and Lewis acidic B with spatial intimacy enables a highly selective nitrile-to-secondary imine transformation.

## Introduction

**Fig. 1:**
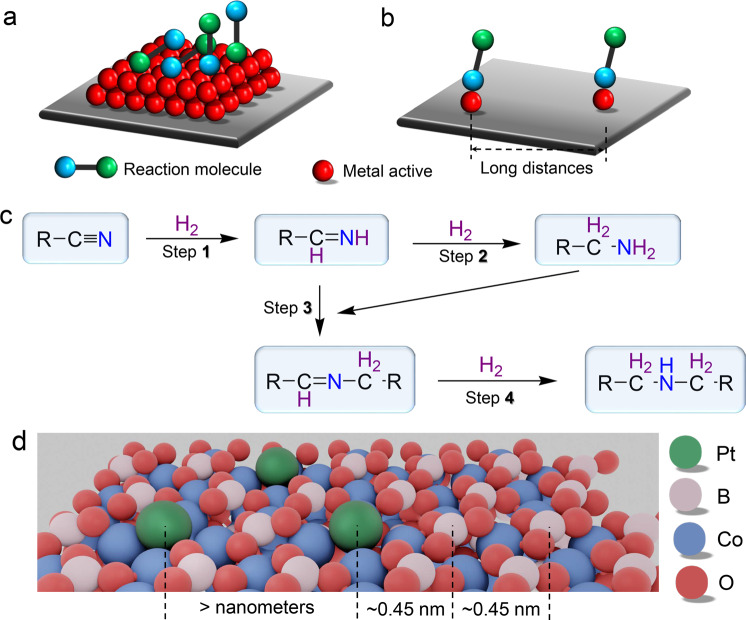
Illustration of the spaital distribution of reactants on various catalytic active sites. Spatial configuration of reactants with binary functional groups on **a** metal nanoparticles, and **b** metal single-atom catalyst sites. **c** Possible pathways of nitrile hydrogenation. **d** Spatial intimacy of binary active-sites of single-atom Pt sites and interfacial Lewis acidic B.

The ideal scenario for heterogeneous catalysis is to solely proceed to a single product with high activity and stability^1^. However, rare cases can realize this ultimate target, especially for catalytic selectivity. Generally, several pathways or elementary steps controlled by thermodynamics and kinetics could sequentially and/or parallelly take place on nano-scaled catalyst surfaces owing to the possible interaction of metal sites with each functional group (Fig. 1a), therefore resulting in a poor selectivity. Recently, reducing the size of catalytically active components has been demonstrated to effectively improve the catalytic selectivity by controlling the adsorption events on active sites of catalysts (Fig. 1b). Especially, the metal single-atom catalysts (SACs) have exhibited the improved catalytic activity and selectivity for various important reactions, including CO_2_ transformation, water–gas shift reaction, semi-hydrogenation of acetylene, and hydrogenation of nitroarenes^2–4^.

However, many catalytic reactions generally require multiple cascaded steps with several reactant molecules and/or intermediates on their respective active sites. As a typical cascaded reaction, the selectively sequential hydrogenation–condensation of nitriles into secondary imines is highly desirable since it represents a green and cost-effective one-step process to replace the previously traditional methods^5–7^. Generally, four possible distinct processes are involved (Fig. 1c): (1) hydrogenation of nitriles into primary imines, (2) and then into primary amines, (3) nucleophilic attack on the electron-deficient carbon of primary imines by primary amines to produce secondary imines, and (4) undesired over-hydrogenation of secondary imines^8,9^. Previous reports have proved that all these elementary steps take place on catalysts surface^10^. Thus, hydrogenation of several nitrile molecules can simultaneously occur on the surface of a metal nanoparticle, which is beneficial for the condensation process to give secondary imines. However, severe over-hydrogenation of secondary imines to secondary amines is usually inevitable due to the strong adsorption of secondary imines on metal nanoparticles^11–15^. While, the primary amines were also obtained in the presence of acids or alkaline additives, which could suppress the occurrence of Step 3^16–19^. By examining plenty of literature, the chemoselectivity transformation of nitrile into secondary imines is still lacked fundamental understandings and faces great scientific challenges. Thus, to design a heterogeneous catalyst with high activity and selectivity for the selective nitrile-to-imine transformation is highly needed.

Theoretically, the metal SACs might avoid the over-hydrogenation of secondary imines due to the weak interaction between reactants and small size metals, especially those of sub-nanometer clusters and SACs^20,21^. However, the isolated metal single-atom active sites with the large inter-distances are only occupied by one reactant molecule (Fig. 1b), thereby making the condensation step difficult and inhibiting the occurrence of Step 3. Taking the above analysis into considerations, if another active site is introduced with a well-organized spatial distribution, the metal SACs might break this limitation to achieve the selective nitrile-to-imine transformation. The Lewis basic *N* atom in nitriles and the hydrogenated intermediates have inspired the use of Lewis acidic sites to trap reactants and intermediates through a strong acid–base interaction.

In this work, the atomically dispersed Pt in CoBO_*x*_ nanosheets (Pt_1_/CoBO_*x*_) has been designed to successfully realize the highly selective nitrile-to-imine transformation with high activity and stability in the absence of any additives. The CoBO_*x*_ nanosheets were selected owing to their abundance of the interfacial Lewis acidic B sites to trap nitriles as well as hydrogenated intermediates. In the Pt_1_/CoBO_*x*_ catalyst, three spatial configurations of binary active sites of single-dispersed Pt site and interfacial Lewis acidic B site are illustrated in Fig. 1d: Pt…Pt with a large inter-distance over nanometers; neighboring Pt…B and adjacent B…B site with a distance of ~0.45 nm. Since the strong adsorption of nitriles and intermediates on single-dispersed Pt sites, theoretically, only the latter two couples can allow the successful condensation if considering their spatial configurations. Catalytic mechanism investigations show that the atomically dispersed Pt sites guarantee efficient hydrogen dissociation. Afterward, the efficient hydrogenation of the absorbed nitriles on adjacent Lewis acidic B sites through hydrogen spillover from Pt to B and thereby subsequent condensation (Step 3) are the energetically favorable pathways. Such a catalyst with the spatially organized single-dispersed Pt and Lewis acidic B binary active-sites can yield a >99.9% selectivity and 864 h^−1^ TOF of benzonitrile hydrogenation to corresponding secondary imines.

## Results

### Preparation and characterization of the Pt_1_/CoBO_*x*_ catalyst

**Fig. 2:**
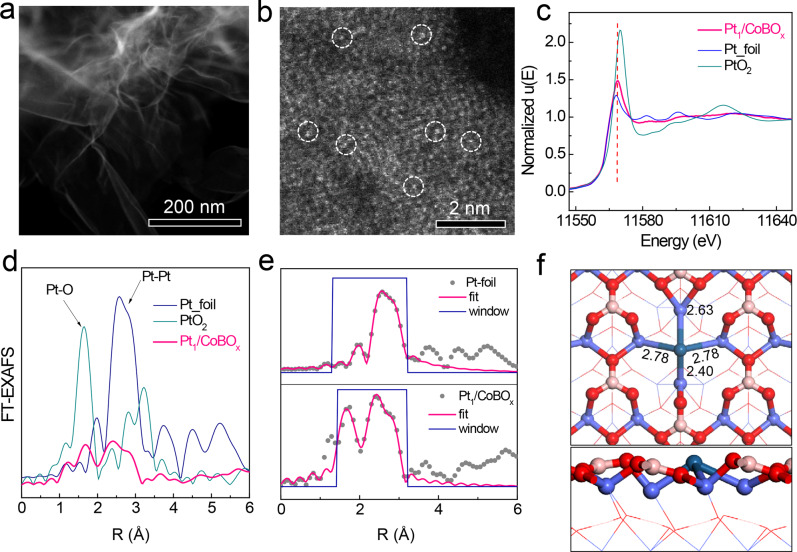
Characterizations of the Pt_1_/CoBO_*x*_ catalyst. **a** Darkfield TEM and **b** HAADF-STEM images of Pt_1_/CoBO_*x*_. **c** XANES and **d** EXAFS of Pt_1_/CoBO_*x*_, Pt foil, and PtO_2_ powder. (*k*^*3*^ weighted data). **e** Curve fittings of EXAFS data. **f** Calculated model of Pt_1_/CoBO_*x*_. The cyan, blue, red, and pink balls represent the Pt, Co, O, and B atoms, respectively.

The Pt_1_/CoBO_*x*_ catalyst with 0.79 wt% Pt-loading was prepared by a facile wet chemical process. Darkfield transmission electron microscopy (TEM) image showed an ultrathin nanosheet morphology of catalyst without any apparent metallic particles/clusters (Fig. 2a). Meanwhile, energy dispersive spectroscopy revealed the uniform Pt distribution in the nanosheets (Supplementary Fig. 1), indicating the highly dispersed Pt on CoBO_*x*_. Also, the high-angle annular dark-field scanning TEM (HAADF-STEM) image further revealed the atomically isolated Pt species were throughout the whole nanosheets (Fig. 2b).

However, different from the oxidized Pt in previously reported SACs^22–26^, X-ray photoelectron spectrum (XPS) suggested a near metallic state of Pt in Pt_1_/CoBO_*x*_ catalyst (Supplementary Fig. 2). This phenomenon could be attributed to the reductive environment of synthetic solution in the presence of NaBH_4_ as well as the much easier reducibility of Pt^2+^ ions than that of Co^2+^ ions according to their standard reduction potentials. To clearly elucidate the Pt chemical status, X-ray absorption fine spectroscopy (XAFS) was applied. X-ray absorption near-edge structures of Pt K-edge revealed that the white line peak of the Pt_1_/CoBO_*x*_ catalyst located at 11568.4 eV was between those of Pt^0^ (11568.0 eV) and PtO_2_ (11569.8 eV), but very close to that of Pt^0^ (Fig. 2c). Also, the peak area of the white line peak was slightly larger than that of Pt^0^. These results illustrated that the electronic structure of Pt was close to the metallic state, consistent with XPS results.

Extended XAFS (EXAFS) spectra were acquired to provide the chemical environment of Pt. Compared with PtO_2_, the peak of catalyst at ~1.6 Å was labeled as Pt–O bond (Figs. 2d, e). The second peak of catalyst between 2.2 and 3.2 Å was well fitted with Pt–Co scattering path (Table 1 and Supplementary Fig. 3a), rather than Pt–Pt or Pt–B scattering path with large R-factors (Supplementary Table 1 and Supplementary Fig. 3b, c). Thus, the negligible Pt–Pt coordination suggested bare Pt particles/clusters in catalysts. Wavelet transform of EXAFS also showed that EXAFS of this catalyst was mainly contributed by Pt–O, and Pt–Co pathways (Supplementary Fig. 4). The averaged coordination number (CN) of Pt–Co was ~4.7, indicating that one Pt atom was chemically bonded with 4–5 Co atoms with an average bond length of 2.61 Å.

**Table 1:** Structural parameters of catalysts and Pt foil from the EXAFS fittings.

		CN (a.u.)	*R* (Å)	*σ*^2^ (10^−3^)	*R*-factor
Pt-foil	Pt–Pt	12^a^	2.76 ± 0.01	3.9 ± 0.1	0.002
Pt_1_/CoBO_*x*_	Pt–O	1.6 ± 0.8	2.04 ± 0.05	4.0 ± 1.1	0.004
	Pt–Pt	0.2 ± 0.6	2.72 ± 0.16	3.0 ± 2.7	
	Pt–Co	4.7 ± 3.0	2.61 ± 0.03	7.3 ± 4.9	

^a^amp = 0.826 ± 0.04.

CN is the averaged coordination number of each shell.

*R* is half of the averaged distance of the scattering path.

*σ*^2^ is the mean square variation in path length.

Meanwhile, due to the low weight percentage of Pt and the interference of other elements, DFT simulation was used to further confirm the local structure of Pt in the Pt_1_/CoBO_*x*_ catalysts, which could exclude the possibility of wrong structure caused by fitting error. Theoretical models of catalysts were constructed by a Pt atom and Co_3_(BO_3_)_2_(200) slab to examine the XFAS-derived structures. The single Pt atom can be adsorbed on the surface or doped by replacing surface Co, O, B atoms, or one BO_3_ unit (Fig. 2f and Supplementary Fig. 5). Among them, the one Pt-replaced-BO_3_ is the most stable structure with the most negative formation energy (Supplementary Fig. 5). The simulated bond lengths of Pt–Co (2.40–2.78 Å, the average value of 2.65 Å) were highly consistent with the EXAFS fittings (Pt–Co: 2.61 Å). Also, one Pt atom was bonded with four Co atoms herein (Fig. 2f), leaving the top of Pt for binding with oxygen, which was consistent with the fitted CN of Pt–Co (4.7) and Pt–O (1.6, Table 1). Thus, the single-atom Pt bonded with 4–5 Co atoms on CoBO_*x*_ could be verified by combining experimental and DFT data.

The structural features of Pt_1_/CoBO_*x*_, revealed from both the experimental and theoretical results, strongly suggest the formation of the unique local configuration of single-atom Pt with four Co atom (Pt_1_–Co_4_ sites) in the catalyst. However, those Co atoms with the saturated coordination states are located at the subsurface or the second atom layer (Fig. 2f). Thus, Co atoms in the clusters are unsuitable to serve as the active sites due to the large steric hindrance and the saturated coordination environment, thereby denying the accessibility of nitriles onto Co sites and leaving only Pt sites interacting with nitriles. However, the condensation (Step 3 in Fig. 1c) is also difficult to occur on two atomically dispersed Pt sites owing to their large distance. Luckily, the singly dispersed Pt site is surrounded by several Lewis acidic B sites, giving a distance of 0.45 nm (Fig. 1d and Fig. 2f). Besides, all interfacial Lewis acidic B sites are adjacent with a short distance of 0.45 nm. Generally, Lewis acid is the catalyst for the condensation step^27,28^. Therefore, combining the atomically dispersed Pt sites for H_2_ activation and the interfacial Lewis acidic sites for condensation, the dual active sites in Pt_1_/CoBO_*x*_ with three spatial configurations might break the limitation of adsorption in the metal SACs and catalytically transform nitriles into secondary imines.

### Catalytic performance of Pt_1_/CoBO_*x*_ catalyst

**Fig. 3:**
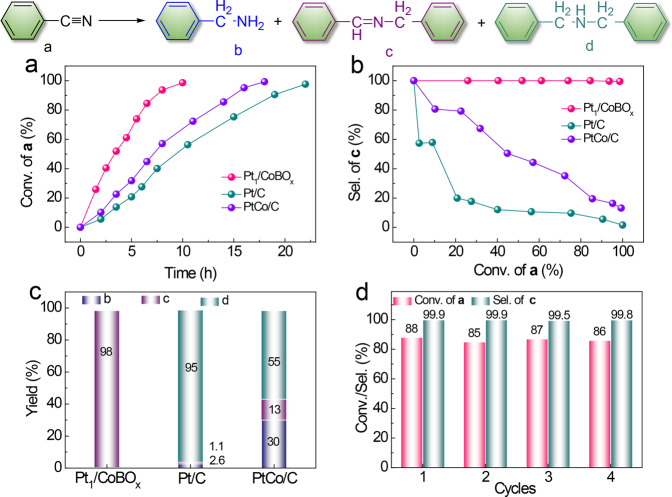
Catalytic performance of the Pt_1_/CoBO_*x*_, Pt/C, and PtCo/C catalysts. **a** Time course of benzonitrile conversions. **b** Benzonitrile conversion *vs*. selectivity of secondary imines. **c** Final yield of various products. **d** Stability of Pt_1_/CoBO_*x*_. Reaction conditions: benzonitrile (1 mmol), isopropanol (2 mL), catalysts (5 mg), 90 °C, and 1 MPa H_2_. The reaction time of the stability test was 8 h.

Inspired by such well-organized spatial distribution of binary active sites in Pt_1_/CoBO_*x*_, hydrogenation of benzonitrile was selected as a model reaction and optimized at 90 °C and 1 MPa H_2_ in isopropanol. As a reference catalyst, Pt nanoparticles on carbon black (Pt/C, 0.8 wt%, 4.6 ± 0.7 nm) were prepared by coprecipitation method (Supplementary Fig. 6). The obtained Pt/C catalyst yielded a 97.6% conversion of benzonitrile after 22 h (Fig. 3a) with a TOF value of 569 h^−1^ based on each exposed Pt atom. However, serious over-hydrogenation was observed (Fig. 3b). The final product was dibenzylamine (95%) with very low yields of secondary imines *N*-benzylidenebenzylamine (1.1%) and benzylamines (2.6%) (Fig. 3c).

Comparatively, the Pt_1_/CoBO_*x*_ catalysts delivered much higher activity under identical conditions, reaching 98.4% conversion of benzonitrile after 10 h (Fig. 3a). Meanwhile, the TOF value based on each Pt atom (864 h^−1^) was 1.5 times higher than that of Pt/C (569 h^−1^). Most importantly, the selectivity of *N*-benzylidenebenzylamine was significantly improved to 99.9% (Fig. 3b). While, the yields of benzylamine and *N*-benzylideneamine were below the detection limitations of gas chromatography–mass spectrometry (GC–MS) (Fig. 3c). In addition, the Pt_1_/CoBO_*x*_ catalysts also exhibited the preserved performance at least for four consecutive cycles (Fig. 3d). After the reaction, the Pt_1_/CoBO_*x*_ catalyst could be easily recycled by centrifugal separation and reused for the next cycle without any treatment. Also, the concentration of Pt in the reaction solution was 30 ppm by inductively coupled plasma optical emission spectrometer analysis. Thus, only 0.5 wt% of Pt (relative to the initial Pt amount in Pt_1_/CoBO_*x*_) was leached from the Pt_1_/CoBO_*x*_ catalyst after four cycles of the repeatedly catalytic reactions, revealing a very low metal loss and highly structural robustness of catalysts during cycling. Meanwhile, the spent catalysts reserved the initial morphological features. Therefore, the structural robustness of Pt_1_/CoBO_*x*_ could indicate their catalytic stability during the hydrogenation of benzonitrile (Supplementary Fig. 7).

### Mechanism investigations

Compared with the Pt/C catalyst, Pt_1_/CoBO_*x*_ exhibited the greatly improved catalytic selectivity of imines as well as activity. Previous reports have proved that alloy nanoparticles can drive the product distribution shift, especially for the introduction of Co for hydrogenation of nitriles^29–31^. However, the unique geometrical configuration of Pt_1_–Co_4_ sites in Pt_1_/CoBO_*x*_ suggests that the introduction of Co does not play a critical function in the selective transformation of benzonitriles to secondary imines. The detailed reasons are listed as follows: (1) the huge steric hindrance on Pt_1_–Co_4_ sites makes it difficult to bind with two reactant molecules simultaneously; (2) Co atoms in Pt_1_–Co_4_ sites are located in the subsurface rather than the catalyst surface; (3) Co atoms are saturated by O and Pt atoms, leading to the blocked sites to bind with other reactant molecules. However, the integration of single-atom Pt and four Co atoms can improve the capability for hydrogen activation^32,33^.

Objectively, it is difficult to precisely synthesize the Pt_1_–Co_4_ clusters on other supports and experimentally demonstrate their influence on the catalytic performance. Herein, the PtCo/C catalysts with 1:4 atomic ratio of Pt:Co and 1 wt% Pt-loading (Supplementary Fig. 8a) were chosen as the nearest approximation model catalyst to understanding the roles of Co incorporated with Pt on the catalytic behavior. As shown in Supplementary Fig. 8b, the measured lattice spacing of 0.215 nm from the HRTEM image of PtCo/C was smaller than the Pt(111) crystalline plane (0.227 nm), but larger than the Co(111) plane (0.205 nm), which indicated the successful formation of the alloyed PtCo nanoparticles on the surface of the carbon. As shown in Fig. 3, the PtCo/C catalyst exhibited the improved catalytic activity compared with the Pt/C catalyst, which was still much slower than that of the Pt_1_/CoBO_*x*_ catalyst. Most importantly, the selectivity of PtCo/C towards secondary imines *N*-benzylidenebenzylamine was similar to Pt/C and greatly lower than that catalyzed by Pt_1_/CoBO_*x*_ at the end of the reaction under the same conditions. While the selectivity of benzylamines was increased to 30% for the PtCo/C catalysts. Obviously, integration of Co and Pt did not shift the selectivity of secondary imines herein. Therefore, this experimental evidence proved that the Pt_1_–Co_4_ sites in Pt_1_/CoBO_x_ were not the main factor for the selective transformation of benzonitriles into secondary imines.

**Fig. 4:**
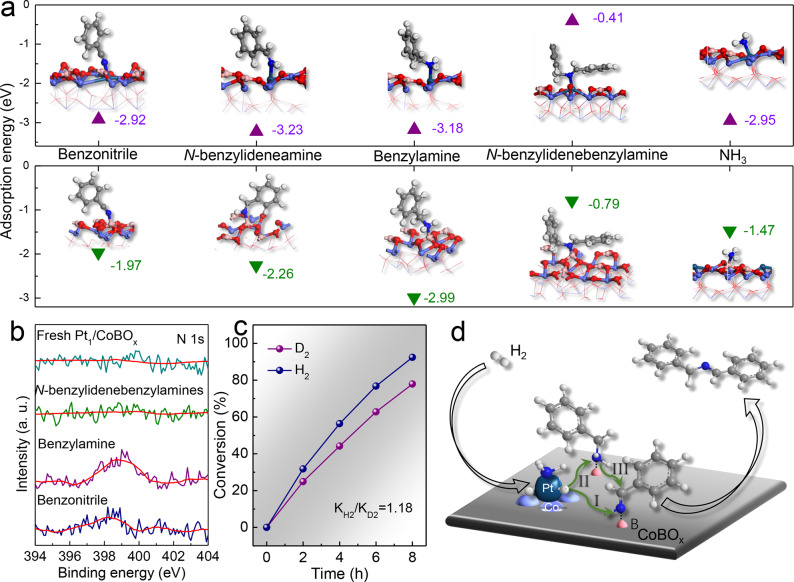
Catalytic mechanism analysis. **a** Adsorption of benzonitrile, dibenzylamine, benzylamine, *N*-benzylidenebenzylamine, and NH_3_ on catalysts. Cyan: Pt, Light blue: Co, Red: O, Pink: B, Dark blue: N, Dark grey: C, Light grey: H. **b** XPS analysis of catalysts before and after treatments with various molecules. **c** Primary isotope effects on the catalytic performance of Pt_1_/CoBO_*x*_. **d** Proposed catalytic mechanism. Cyan: Pt, Light blue: Co, Red: O, Pink: B, Dark blue: N, Dark grey: C, Light grey: H.

Actually, the catalytic performance of Pt/C and PtCo/C catalyst experimentally revealed that transformation of nitriles into secondary amines on Pt or PtCo nanoparticles suffered from the severe over-hydrogenation into secondary amines, similar to previous reports^34,35^. It’s generally attributed to the strong adsorption of imines on the large metal particles^36^. Thus, the hydrogenation pathway of Pt_1_/CoBO_*x*_ with the simultaneously enhanced catalytic activity and selectivity is different from the particle counterparts. To examine the importance of the spatial distribution of binary active sites as well as their proximity, density functional theory (DFT) calculations were performed to examine the adsorption behavior of various molecules on Pt_1_/CoBO_*x*_. Benzonitrile is adsorbed on the Pt top with an adsorption energy of −2.92 eV via *N* atom interaction with Pt (Fig. 4a and Supplementary Fig. 9). The respective adsorption energies of *N*-benzylideneamine and benzylamine on Pt_1_ site are −3.23 and −3.18 eV with similar adsorption configurations. Such strong adsorption of intermediate reveals that the nucleophilic attack in Step 3 would not be efficient in the reaction solution via desorption from the catalyst surface. This could be also confirmed from the undetected intermediates during the reaction. Therefore, the effectiveness of Step 3 will strongly depend on the proximity of two Pt atoms occupied by respective primary amine and primary imine. Unfortunately, the long average inter-distance between two surface Pt atoms (Fig. 2b), makes the nucleophilic attack in Step 3 difficult, impeding the condensation to form secondary imines on the atomic Pt sites.

While the Pt_1_/CoBO_*x*_ catalysts did efficiently catalyze the selective nitrile-to-secondary imine hydrogenation (Fig. 3). Thus, alternative active sites in Pt_1_/CoBO_*x*_ catalyst should provide the energetically favorable pathways to promote this reaction. Considering the conventional Lewis acid-catalyzed process, the abundant Lewis acidic B sites on CoBO_x_ (Supplementary Fig. 10a, b) make us conjecture their highly possible acid-base interaction with reactants via Lewis basic *N* and catalyze the Step 3^19,37,38^. DFT calculations verified such a strong acid (B sites)–base (benzonitrile) interaction via a tilted configuration with an adsorption energy of −1.97 eV (Fig. 4a and Supplementary Figure. 11). The respective adsorption energies of *N*-benzylideneamine and benzylamine on B sites were −2.26 eV and −2.99 eV. Due to the abundant surface B sites, *N*-benzylideneamine and benzylamine on two adjacent B sites with a close intimacy of ~0.45 nm have great opportunities to directly condensate into secondary imines and leave one NH_3_ on B site. Expectedly, *N*-benzylidenebenzylamine exhibits very weak adsorption on CoBO_*x*_ (−0.79 eV, Fig. 4a) owing to the strong intermolecular tension and large steric hindrance, avoiding the over-hydrogenation of imines. NH_3_ left on B sites with an adsorption energy of only −1.47 eV can be replaced by benzonitrile for the next cycle. Also, the neighboring *N*-benzylideneamine and benzylamine on adjacent B and single-dispersed Pt sites with a close distance of ~0.45 nm also can catalyze Step 3 with weaker interaction with *N*-benzylidenebenzylamine (−0.41 eV). However, the much stronger adsorption of NH_3_ on Pt (−2.95 eV) indicates the difficulty in releasing the active sites for the next cycle, compared to that on B sites, suggesting the energetically favorable pathways on the adjacent Lewis acidic B sites.

To experimentally confirm their adsorption behaviors, the Pt_1_/CoBO_*x*_ catalysts were treated by benzonitrile, benylamine and *N*-benzylidenebenzylamines at 90 °C for 1 h, respectively. Their interactions were monitored by the XPS N1s peak (Fig. 4b). Similar to the fresh catalyst, Pt_1_/CoBO_*x*_ treated by *N*-benzylidenebenzylamines showed a bare N1s signal. While the strong N1s peaks were detected for catalysts treated by benzonitrile and benzylamine. Thus, revealed from the XPS and DFT data, the Pt_1_/CoBO_*x*_ catalyst showed a weak interaction with secondary imines but strong adsorption of nitriles and primary amines.

Meanwhile, the CoBO_*x*_ nanosheets (Supplementary Fig. 12) were prepared in the absence of the Pt precursor and their catalytic performance was evaluated to further identify the function of single-atom Pt. As shown in Supplementary Fig. 13, the CoBO_*x*_ nanosheets exhibited no catalytic activity for the hydrogenation of benzonitrile even after 20 h under the same reaction conditions. Therefore, the atomically dispersed Pt sites are recognized to activate H_2_. Despite Pt sites might be occupied by reactants (nitriles) or intermediates (primary imines/amines, and NH_3_), the single-dispersed Pt bonded with Co atom can allow the accessibility of H_2_ due to its small size. Different from the heterolytic dissociation on single-dispersed metal catalyst^26^, herein, hydrogen would undergo homolytic dissociation on the Pt_1_/CoBO_*x*_ catalyst into active hydrogen species due to the near metallic state of Pt as well as the presence of vicinal two metal atoms Pt and Co. The kinetic isotope effect with the use of D_2_ in the benzonitrile hydrogenation was used to examine the proposed hydrogen homolytic dissociation pathway. The hydrogenation was slightly slowed down by a factor of 1.18 as a result of the zero-point energy difference between isotopic isomers, similar to the conventional metal catalysts (Fig. 4c), indicating the homolytic dissociation of hydrogen on the surface of Pt_1_/CoBO_*x*_ catalysts^26^. Then, the activated hydrogen species are spilled from the single-dispersed Pt to B sites for hydrogenation. This spillover was confirmed by the color change of the mixture of WO_3_ and Pt_1_/CoBO_*x*_ under 1 MPa H_2_ at 30 °C for 0.5 h (Supplementary Fig. 14).

Therefore, both experimental and theoretical results stated the importance of the spatial intimacy of single-dispersed Pt sites and interfacial Lewis acidic B sites for such a highly selective nitrile-to-secondary imine hydrogenation. Strong adsorption of primary amines on both single-atom Pt and Lewis acidic B sites can effectively avoid the release of by-product amines. While weak adsorption of secondary imines on Pt_1_/CoBO_x_ can suppress their over-hydrogenation. As illustrated in Fig. 3d, our approach includes several processes as the elementary steps of the catalytic cycle: the homolytic hydrogen activation on single-dispersed Pt site in the presence of Pt–Co bond (I); the spillover of the active hydrogen species from Pt to Lewis acidic B sites for hydrogenating benzonitrile into *N*-benzylideneamine and benzylamine (II); their condensation into secondary imines on adjacent Lewis acidic B sites (III); and easy desorption of secondary imines from the catalyst surface due to the strong intermolecular tension and steric hindrance of imines (IV). The much stronger adsorption of nitriles than that of NH_3_ on the B site guarantees the recovery of active sites for the next cycle. The Pt_1_/CoBO_*x*_ catalyst also showed good performance for several substituted nitriles (Table 2).

**Table 2:** Hydrogenation of various substituted nitriles^a^.

^a^Reaction conditions: substrates (0.5 mmol), isopropanol (2 mmol), catalysts (5 mg), 90 °C, 1 MPa H_2_ and 6 h.

^b^100 °C and 18 h.

## Discussion

We have demonstrated the importance of the spatial intimacy of the binary active sites in a well-designed atom-dispersed metal catalyst for the complex catalytic reactions, which may be very different from the general metal SAC catalysts as well as the metal nanoparticle catalysts. The Pt_1_/CoBO_*x*_ catalysts with the spatially well-organized binary active sites of the single-dispersed Pt sites and interfacial Lewis acidic B sites have been rationally designed and dedicatedly synthesized to realize the highly selective nitrile-to-secondary imine hydrogenation. The long inter-distance between two single-dispersed Pt sites can enable the hydrogenation but inhibit the consequent condensation due to the spatial limitation. While, the adjacent interfacial Lewis acidic B sites and the neighboring single-dispersed Pt and interfacial Lewis acidic B sites with the intimate distances of ~0.45 nm can guarantee both the hydrogenation and condensation to successfully achieve this selective transformation. Experimental results and DFT calculations suggest the sequence of the hydrogen activation at single-dispersed Pt sites, hydrogen spillover from Pt to CoBO_*x*_ supports, nitrile hydrogenation and condensation at the adjacent Lewis acidic B sites on CoBO_*x*_ provides the energetically favorable pathways for this highly selective nitrile-to-imine transformation. This strategy provides insights into this previously difficult transformation by the rationally designed heterogeneous catalysts.

## Methods

### Preparation of Pt_1_/CoBO_*x*_ catalysts

The Pt_1_/CoBO_*x*_ catalysts were prepared by a wet chemical process. Initially, 3 mmol of Co(NO_3_)_2_·6H_2_O and 0.015 mmol of (NH_3_)_4_Pt(NO_3_)_2_ were completely dissolved in 280 mL H_2_O. Then, 20 mL of freshly prepared NaBH_4_ solution (0.375 M) was quickly added under vigorous stirring at room temperature for 1 h. After 2 h aging, the Pt_1_/CoBO_*x*_ catalysts were alternatively washed by water and ethanol for three times. Finally, the Pt_1_/CoBO_*x*_ catalysts were dried at 60 °C for 6 h.

### Preparation of Pt/C and PtCo/C catalysts

The Pt/C catalysts were prepared by the chemical coprecipitation method. Firstly, 200 mg of carbon black were washed by hydrochloric acid (1 M) and acetone, and then dispersed in 20 mL of H_2_O. The dispersion solution was stirred for 1 h at room temperature after adding 5 mL of (NH_3_)_4_Pt(NO_3_)_2_ solution (Pt: 0.32 mg mL^−1^). Then, 15 mL of aqueous urea solution (10 mg mL^−1^) was added into the above solution. Afterward, the reaction temperature was increased to 70 °C for 2 h. Next, 10 mL of the ice-cold fresh NaBH_4_ solution (1 mg mL^−1^) was added for another 0.5 h reaction at room temperature. Finally, the Pt/C catalysts were collected after thorough washing and drying at 60 °C.

The PtCo/C catalyst was prepared by the same process only by changing the 5 mL of (NH_3_)_4_Pt(NO_3_)_2_ solution to 5 mL of (NH_3_)_4_Pt(NO_3_)_2_ and Co(NO_3_)_2_ mixture solution.

### Characterizations

TEM studies were conducted with a Hitachi HT-7700 transmission electron microscope with an accelerating voltage of 120 kV. XPS spectra was acquired using a Thermo Electron model K-Alpha with Al *K*_*α*_ as the excitation source. High-resolution TEM was conducted on a Titan Cubed Themis G2 300 (FEI) aberration-corrected scanning transmission electron microscope.

### Catalytic hydrogenation

The nitrile hydrogenation was carried out in a stainless-steel autoclave equipped with the pressure control system. For a typical catalytic reaction, 1 mmol of benzonitrile and 5 mg of catalysts were mixed in 2 mL of isopropanol. The reactions were performed after charging with 1 MPa H_2_ at 90 °C. After the reaction, the products were analyzed by GC–MS and GC.

### Adsorption experiments

The adsorption experiments were operated in glass bottles (10 mL). Initially, 5 mg of Pt_1_/CoBO_*x*_ and 1 mmol of adsorbates (benzonitrile, N-benzylidenebenzylamine, or benzylamine) were mixed in 2 mL of isopropanol. The mixtures were stirred at 90 °C for 2 h. Afterwards, the treated Pt_1_/CoBO_*x*_ catalysts were collected by centrifugalization, washed by ethanol for three times to remove the un-adsorbed molecules, and dried by vacuum for 10 h. Finally, the treated Pt_1_/CoBO_*x*_ catalysts were examined by XPS.

### Cyclic experiments

The cyclic experiments were also carried out in a stainless-steel autoclave equipped with the pressure control system. For a typical catalytic reaction, 1 mmol of benzonitrile and 5 mg of catalysts were mixed in 2 mL of isopropanol. The reactions were performed after charging with 1 MPa H_2_ at 90 °C after 8 h. After the reaction, the Pt_1_/CoBO_*x*_ catalysts were collected by centrifugalization and used for the next cycle without any treatments. The products were analyzed by GC.

## Supplementary information

Supplementary Information

Peer Review File

## Data Availability

The authors declare that the main data supporting the findings of this are available within the article and Supplementary information from the corresponding author upon reasonable request. Source data are provided with this paper.

## Source data

Source Data
