# Supplementary material for: Spatial intimacy of binary active-sites for selective sequential hydrogenation-condensation of nitriles into secondary imines
Source: Nat Commun. 2021 Jun 7;12:3382. doi: 10.1038/s41467-021-23705-9 (PMC8184996; doi:10.1038/s41467-021-23705-9)

Spatial intimacy of binary active-sites for selective sequential hydrogenation-condensation of nitriles into secondary imines

Sai Zhang[1*], Zhaoming Xia[2*], Yong Zou[2], Mingkai Zhang[2] & Yongquan Qu[1,2]

[1] *Key Laboratory of Special Functional and Smart Polymer Materials of Ministry of Industry and Information Technology, School of Chemistry and Chemical Engineering, Northwestern Polytechnical University, Xi'an, 710072, China*

[2] *Center for Applied Chemical Research, Frontier Institute of Science and Technology, Xi'an Jiaotong University, Xi'an, 710049, China.*

*These authors contributed equally to this work

Correspondence and requests for materials should be addressed to Qu Y. Q. (Email: yongquan@nwpu.edu.cn)

Supplementary Methods

XAFS Experiment and Data Processing. XAFS measurements at Pt L3-edge in both transmission (for Pt foil) and fluorescence (for samples) mode were performed at the BL14W1[1] in Shanghai Synchrotron Radiation Facility (SSRF). The electron beam energy was 3.5 GeV and the stored current was 230 mA (top-up). A 38-pole wiggler with the maximum magnetic field of 1.2 T inserted in the straight section of the storage ring was used. XAFS data were collected using a fixed-exit double-crystal Si(111) monochromator. A Solid detector was used to collect the fluorescence signal, and the energy was calibrated using Pt foil. The photon flux at the sample position was 2.6×10^{12} photons per second.

The raw data analysis was performed using IFEFFIT software package according to the standard data analysis procedures[2]. The spectra were calibrated, averaged, pre-edge background subtracted, and post-edge normalized using Athena program in IFEFFIT software package. The Fourier transformation of the k^3-weighted EXAFS oscillations, $k^3\cdot\chi(k)$, from k space to R space was performed over a range of 3–12 Å^{-1} to obtain a radial distribution function. And data fitting was done by Artemis program in IFEFFIT.

DFT calculation. Spin-polarized DFT calculations were carried out using the plane wave basis Vienna ab initio simulation package (VASP) [3,4] with the electron exchange-correlation potential treated by the generalized gradient approximation (GGA) in the form of Perdew-Burke-Ernzerhof (PBE) functional[5]. DFT-D3 correction method of Grimme[6] was used to describe the Van der Waals interaction. The DFT + U methodology was used to treat the on-site Coulomb and exchange interaction of the strongly localized Co 3d electrons with an

effective U = 2.0 eV[7]. The energy cutoff was 400 eV. For the geometry relaxation, a criterion of 0.02 eV $Å^{-1}$ on forces was used. The convergence criterion for the energy was 10^{-5} eV. Brillouin zone integration was sampled with the 3×3×1 Monkhorst-Pack mesh k-points for surface calculations, and 1×1×1 Gamma point for molecular calculation[8].

The (200) plane of $CoBO_x$ was selected as the theoretical model. The slab models in a (2x2) supercell contained two Co-layers and two BO_3-layers, with the top Co-layer and BO_3-layer being relaxed and the other layers being fixed. All the slabs were separated by a 20 Å vacuum layer.

Structure simulation of the $Pt_1/CoBO_x$ catalyst. Theoretically, single atom Pt can bond with $CoBO_x$ supports *via* adsorption on the surface or replaced the surface atom or group. Thus, there are 8 possible configurations.

The ad-1, ad-2, ad-3 and ad-4 represent that single atom Pt adsorbed on the 4 sites marked in Supplementary Figure 5a, respectively. The formation energy is calculated by the follow Supplementary Equation 1:

$$Ef=E_{tot}-E_{Slab}-E_{Pt\text{-}bulk} \qquad \textbf{Supplementary Equation 1}$$

Where E_{tot} is the total energy of Slab model with one Pt atom adsorbed on it; E_{Slab} is the energy of Slab model without any Pt atoms; $E_{Pt\text{-}bulk}$ is the energy per Pt atom in the most stable bulk phase Pt (FCC).

The replace-m (m=Co, B, O_{2f}, O_{3f}) represent the configurations that one m atom (Supplementary Figure S5b) is replaced by one Pt atom. The formation energy is calculated by the follow Supplementary Equation 2:

$$Ef=E_{tot} - E_{Slab} - E_{Pt\text{-}bulk} + E_m$$ **Supplementary Equation 2**

Where E_m is the energy per m atom in the most stable bulk phase (m=Co, B), or the energy per O in O_2 molecule (m=O_{2f}, O_{3f}).

The replace-BO_3 represent the configuration that one BO_3 group is replaced by one Pt atom. The formation energy is calculated by the follow Supplementary Equation 3:

$$Ef=E_{tot} - E_{Slab} - E_{Pt\text{-}bulk} + (E_{BO3H3} - 3/2E_{H2})$$ **Supplementary Equation 3**

Where E_{BO3H3} is the energy of $B(OH)_3$ molecule; E_{H2} is the energy of H_2 molecule.

The formation energies are related to the stability of each configuration. Among which, replace-BO_3 shows the most negative formation energy. It indicates a big possibility that Pt atoms will replace the surface BO_3 groups in a reductive and relatively high temperature environment.

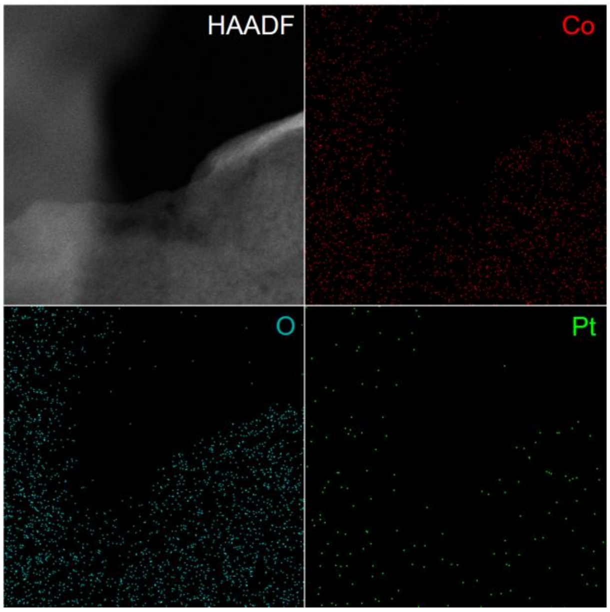

Supplementary Figure 1 | Characterization of the $Pt_1/CoBO_x$ catalyst.

Energy dispersive spectrometer mapping of the $Pt_1/CoBO_x$ catalyst.

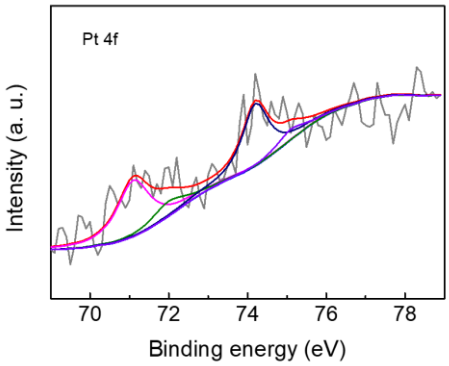

Supplementary Figure 2 | XPS analysis of the $Pt_1/CoBO_x$ catalyst.

The XPS analysis of Pt 4f for the $Pt_1/CoBO_x$ catalyst.

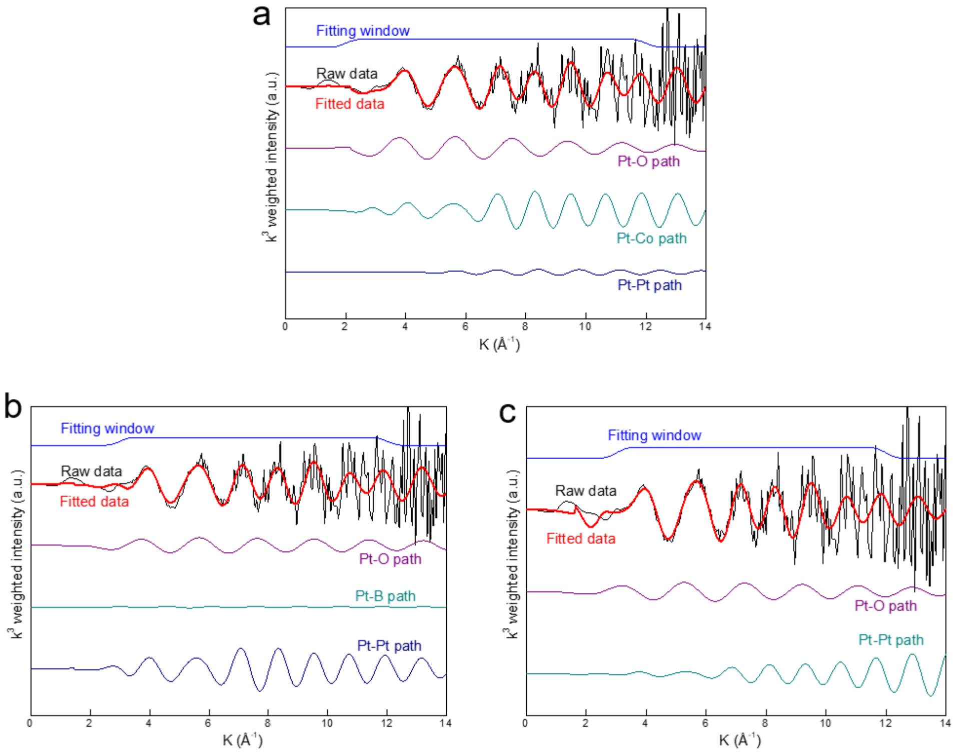

Supplementary Figure 3 | EXAFS spectra of the $Pt_1/CoBO_x$ catalyst.

The k^3-weighted k plot of the raw data of $Pt_1/CoBO_x$, the fitted data, (**a**) Pt-O, Pt-Co, Pt-Pt (Table 1), (**b**) Pt-O, Pt-B, Pt-Pt (Supplementary Table 1, Method 1) and (**c**) Pt-O, Pt-Pt (Supplementary Table 1, Method 2) calculated by Feff with the fitted parameters.

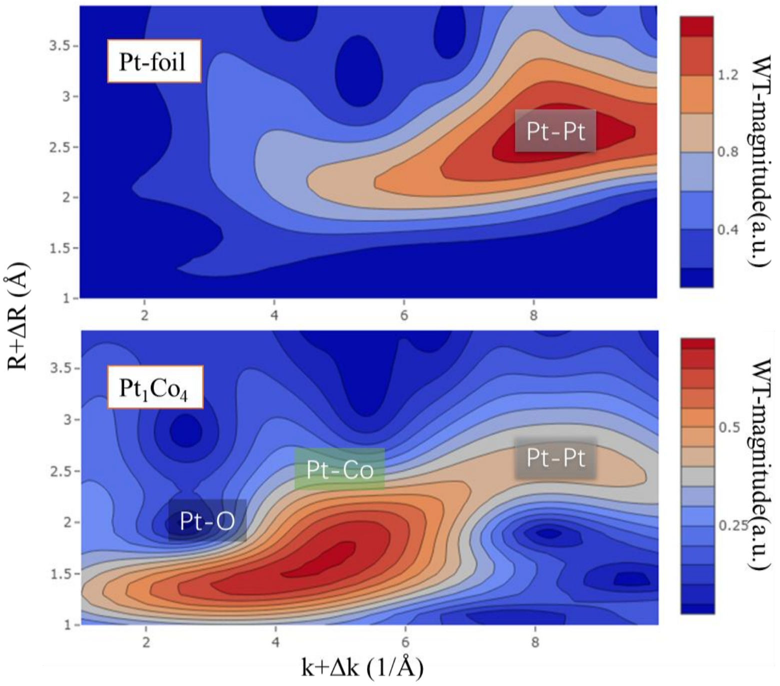

Supplementary Figure 4 | Contour map of Wavelet transformed EXAFS signal.

Morlet wavelet. σ=10, η=0.5.[9] Co has a smaller atomic number than Pt. Thus, the contribution of Pt-Co scattering pathway appears in the smaller k-range than that of Pt-Pt scattering pathway in the wavelet map.

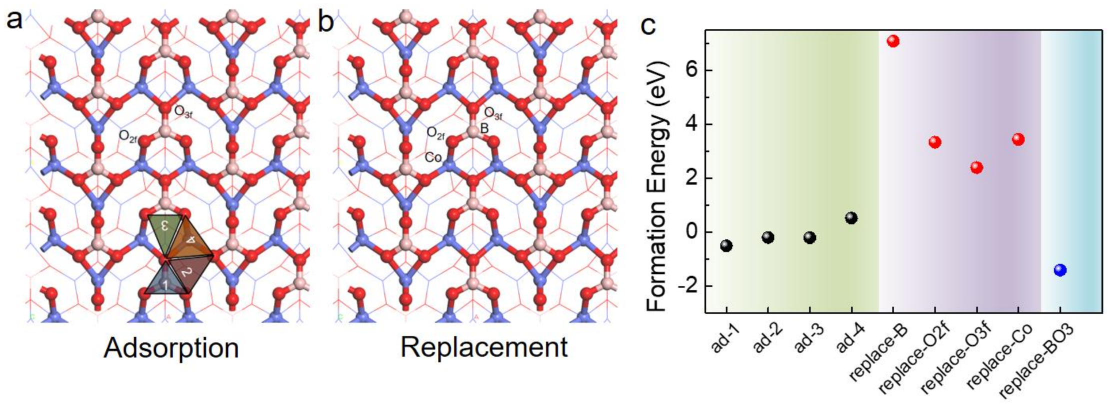

Supplementary Figure 5 | DFT simulation the structure of $Pt_1/CoBO_x$.

(a) The possible adsorption sites of single atom Pt on $Co_3(BO_3)_2$(200). (**b**) The possible replaced sites of single atom Pt on $Co_3(BO_3)_2$(200). (**c**) The summary of formation energy of single atom Pt *via* various models.

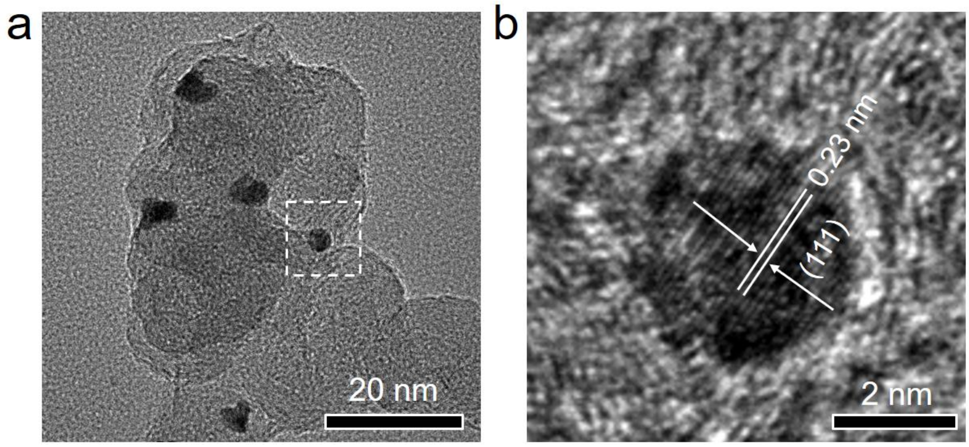

Supplementary Figure 6 | Characterization of the Pt/C catalyst.

(**a**) TEM and (**b**) HRTEM image of the Pt/C catalyst.

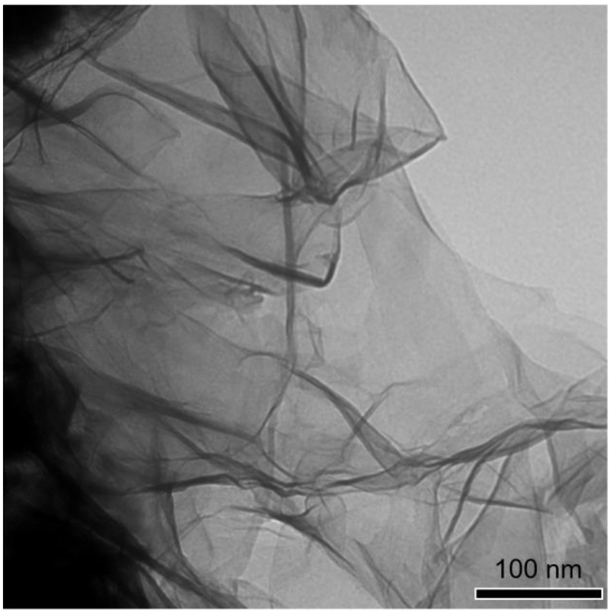

Supplementary Figure 7 | Characterization of the used $Pt_1/CoBO_x$ catalyst.

TEM image of used the $Pt_1/CoBO_x$ catalyst.

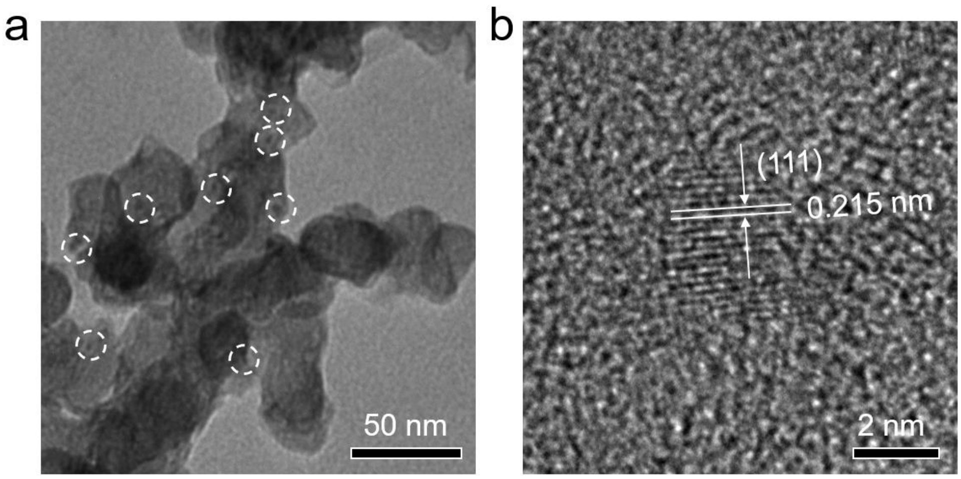

Supplementary Figure 8 | Characterizations of the PtCo/C catalyst.

(**a**) TEM and (**b**) HRTEM images of the PtCo/C catalyst.

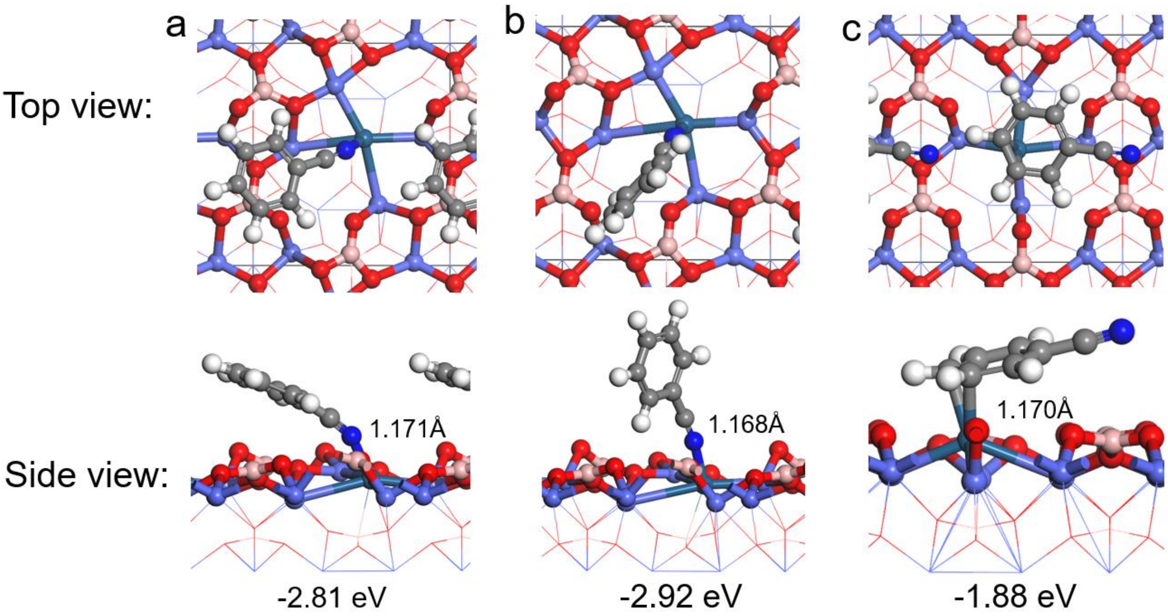

Supplementary Figure 9 | Possible adsorption models of benzonitrile on single-atom Pt.

(**a**) Phenyl group is interacted with the surface by Van Der Waals force, and N atom is bonded with Pt. (**b**) Phenyl group is interacted with the surface by Van Der Waals force, and N and H atoms are bonded with Pt and O atoms, respectively. (**c**) Phenyl group is bonded with Pt atom in the right configuration.

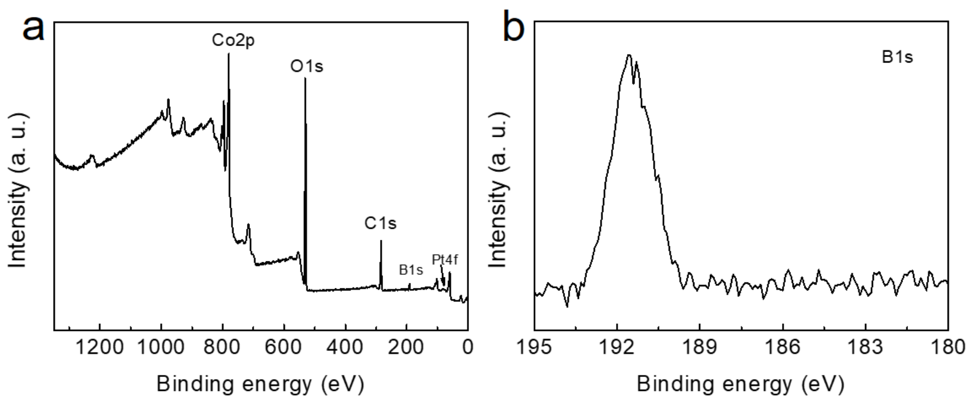

Supplementary Figure 10 | XPS analysis of the $Pt_1/CoBO_x$ catalyst.

(**a**) The XPS profiles of $Pt_1/CoBO_x$ catalyst. (**b**) XPS profiles of B 1s for the $Pt_1/CoBO_x$ catalyst.

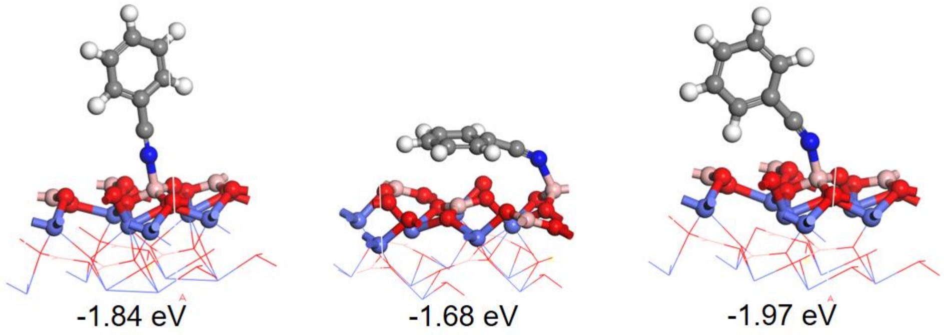

Supplementary Figure 11 | Fitting by Pt-Pt and Pt-B scattering pathways.

The possible adsorption model of benzonitrile on Lewis acidic B sites of $Pt_1/CoBO_x$.

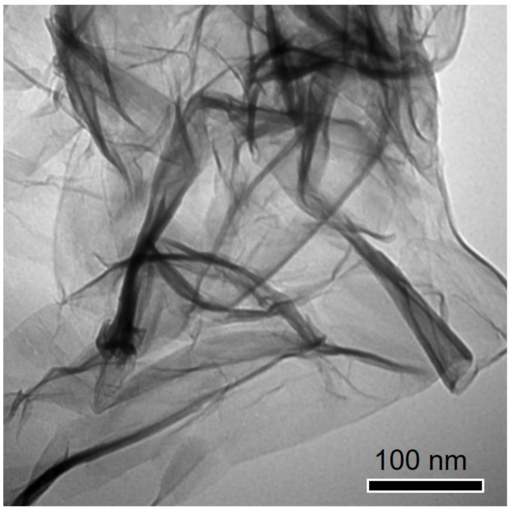

Supplementary Figure 12 | Characterizations of the $CoBO_x$ catalyst.

TEM image of the $CoBO_x$ catalyst.

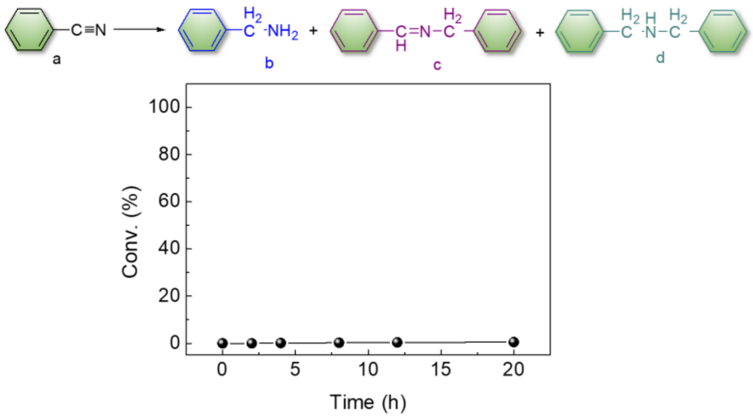

Supplementary Figure 13 | Catalytic performance of $CoBO_x$.

Plot of the conversion of benzonitrile as a function of reaction time for hydrogenation benzonitrile. **Reaction conditions**: benzonitrile (1 mmol), isopropanol (2 mL), $CoBO_x$ (5 mg), 90 °C and 1 MPa H_2.

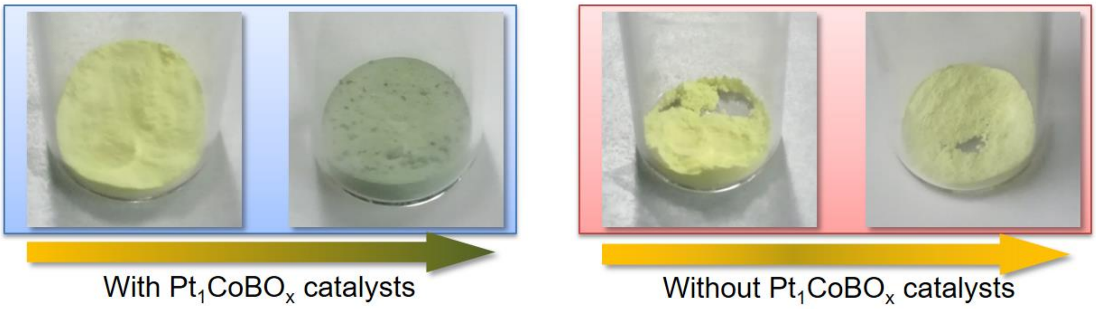

Supplementary Figure 14 | Characterization of hydrogen spillover.

Photograph images of WO_3 treated by hydrogen under 90 °C and 1 MPa H_2 pressure.

Supplementary Table 1 | Fitting by Pt-Pt and Pt-B scattering pathways.

Fitting method		N (a.u.)	R (Å)	σ^2 (10^{-3})	R-factor
Method 1[a]	Pt-O	0.7 ± 0.1	1.99 ± 0.17	3.0 ± 6.4	0.029
	Pt-Pt	6.8 ± 4.3	2.70 ± 0.07	9.1 ± 7.1	
	Pt-B	0.2 ± 0.4	2.69 ± 1.3	3.2 ± 2.7	
Method 2[b]	Pt-O	1.3 ± 2.2	2.00 ± 0.31	5.3 ± 9.4	0.016
	Pt-Pt	5.2 ± 6.5	2.72 ± 0.05	8.7 ± 6.3	

Supplementary Table 2. Catalytic hydrogenation of nitriles by heterogeneous catalysts.

Entry	Catalysts	Substrate : metal	Temperature (^{o}C)	H_2 (MPa)	Time (h)	Product	Ref.
1	Pt/Ni-MOF	200:1	80	0.8	30	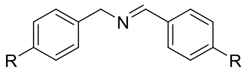	10
2	Pd_1Ni/SiO_2	1732:1	80	0.6	3	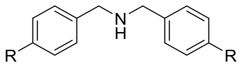	11
3	Pd/C	10:1	80	0.6	7	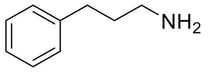	12
4	Ru/C	100:1	100	1	20	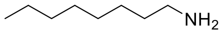	13
5	Pd/C	20:1	40	HCOOH/NEt_3	2	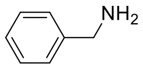	14
6	Pd-$Cu_{0.5}/Fe_3O_4$<br>Pd-$Fe_{0.25}Cu_{0.25}/Fe_3O_4$	10:1	40	NH_3BH_3	1.5	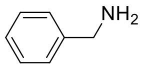<br>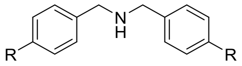	15
7	([Co(OAc)$_2$/Phen@α-Al_2O_3]-800	/	130	4	5	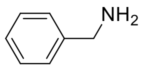	16
8	Co/$Zr_{12}(\mu_3\text{-}O)_8(\mu_3\text{-}OH)_8(\mu_2\text{-}OH)_6$	/	110	4	42	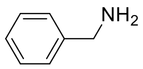	17
9	Ni-phen@TiO_2-1000	/	100	5	20	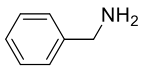	18
10	Co/Zn-powder	/	120	4	15	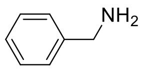	19
11	N-Doped Co@C	/	80	1 mL i-PrOH<br>4 mL i-PrOH	>40<br>>40	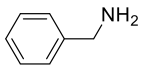<br>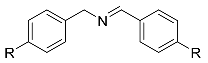	20
12	$Pt_1/CoBO_x$	4100	90	1	9	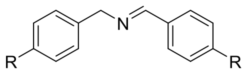	This work

Supplementary Reference

1 Yu, H.; Wei, X.; Li, J.; Gu, S.; Zhang, S.; Wang, L.; Ma, J.; Li, L.; Gao, Q.; Si, R.; Sun, F.; Wang, Y.; Song, F.; Xu, H.; Yu, X.; Zou, Y.; Wang, J.; Jiang, Z.; Huang, Y. The XAFS beamline of SSRF. *Nucl. Sci. Tech.* **2015**, *26*, 50102–050102.

2 Newville, M. IFEFFIT: Interactive XAFS Analysis and FEFF Fitting. *J. Synchrotron Radiat.* **2001**, *8*, 322–324.

3 Kresse, G.; Furthmüller, J. Efficient Iterative Schemes for Ab Initio Total-Energy Calculations Using a Plane-Wave Basis Set. *Phys. Rev. B* **54**, 11169–11186 (1996).

4 Kresse, G.; Hafner, J. Ab-Initio Molecular-Dynamics Simulation of the Liquid-Metal Amorphous-Semiconductor Transition in Germanium. *Phys. Rev. B* 49, 14251–14269 (1994).

5 Perdew, J. P.; Burke, K.; Ernzerhof, M. Generalized Gradient Approximation Made Simple. *Phys. Rev. Lett.* **78**, 1396–1396 (1997).

6 Grimme, S.; Antony, J.; Ehrlich, S.; Krieg, H. A Consistent and Accurate Ab Initio Parametrization of Density Functional Dispersion Correction (DFT-D) for the 94 Elements H-Pu. *J. Chem. Phys.* **132**, 154104 (2010).

7 Jiang, D.; Dai, S. The Role of Low-Coordinate Oxygen on Co3O4(110) in Catalytic CO Oxidation. *Phys. Chem. Chem. Phys.* 13, 978–984 (2011).

8 Monkhorst, H. J.; Pack, J. D. Special Points for Brillouin-Zone Integrations. *Phys. Rev. B* **13**, 5188–5192 (1976).

9 Xia, Z. M.; Zhang, H.; Shen, K. C.; Qu, Y. Q.; Jiang, Z. Wavelet analysis of extended X-ray absorption fine structure data: Theory, application. *CAS OpenIR* **542,** 12–19 (2018).

10 Long, J., Yin, B., Li, Y. & Zhang, L. Selective hydrogenation of nitriles to imines over a

multifunctional heterogeneous Pt catalyst. *AIChE Journal* **60**, 3565-3576 (2014).

11 Wang, H. *et al.* Quasi Pd1Ni single-atom surface alloy catalyst enables hydrogenation of nitriles to secondary amines. *Nat. Commun.* **10**, 4998 (2019).

12 Lévay, K., Tóth, K. D., Kárpáti, T. & Hegedűs, L. Heterogeneous catalytic hydrogenation of 3-phenylpropionitrile over palladium on carbon. *ACS Omega* **5**, 5487-5497 (2020).

13 Hinzmann, A. & Gröger, H. Selective hydrogenation of fatty nitriles to primary fatty amines: Catalyst evaluation and optimization starting from octanenitrile. *Eur. J. Lipid Sci. Technol.* **122**, 1900163 (2019).

14 Vilches-Herrera, M., Werkmeister, S., Junge, K., Börner, A. & Beller, M. Selective catalytic transfer hydrogenation of nitriles to primary amines using Pd/C. *Catal. Sci. Technol.* **4**, 629-632 (2014).

15 Liu, L. *et al.* Pd-CuFe catalyst for transfer hydrogenation of nitriles: Controllable selectivity to primary amines and secondary amines. *iScience* **8**, 61-73 (2018).

16 Chen, F. *et al.* Stable and inert cobalt catalysts for highly selective and practical hydrogenation of C=N and C=O bonds. *J. Am. Chem. Soc.* **138**, 8781-8788 (2016).

17 Ji, P. *et al.* Single-site cobalt catalysts at new $Zr_{12}(\mu_3\text{-}O)_8(\mu_3\text{-}OH)_8(\mu_2\text{-}OH)_6$ metal-organic framework nodes for highly active hydrogenation of nitroarenes, nitriles, and isocyanides. *J. Am. Chem. Soc.* **139**, 7004-7011 (2017).

18 Ryabchuk, P. *et al.* Intermetallic nickel silicide nanocatalyst-A non-noble metal-based general hydrogenation catalyst. *Sci. Adv.* **4**, eaat0761 (2018).

19 Timelthaler, D. & Topf, C. Liquid-phase hydrogenation of nitriles to amines facilitated by a Co(II)/Zn(0) pair: A ligand-free catalytic protocol. *J. Org. Chem.* **84**, 11604-11611 (2019).

20 Long, J., Shen, K. & Li, Y. Bifunctional N-doped Co@C catalysts for base-free transfer hydrogenations of nitriles: Controllable selectivity to primary amines *vs* imines. *ACS Catal.* **7**, 275-284 (2016).

Supplementary Data 1. Feff input file to generate Pt-O path in Supplementary Figure 3.

It is generated using the crystal structure file of PtO_2.

```
* This feff6 file was generated by Demeter 0.9.25
 * Demeter written by and copyright (c) Bruce Ravel, 2006-2016

 * --*--*--*--*--*--*--*--*--*--*--*--*--*--*--*--*--*--*--*--*--*--*--*--
 *    total mu*x=1:    7.208 microns,    unit edge step:    12.304 microns
 *    specific gravity:    8.366
 * --*--*--*--*--*--*--*--*--*--*--*--*--*--*--*--*--*--*--*--*--*--*--*--
 *    normalization correction:        0.00043 ang^2
 * --*--*--*--*--*--*--*--*--*--*--*--*--*--*--*--*--*--*--*--*--*--*--*--

 TITLE PtO2

 HOLE           4      1.0      * FYI: (Pt L3 edge @ 11564 eV, second number is S0^2)
 *                  mphase,mpath,mfeff,mchi
 CONTROL     1          1         1         1
 PRINT         1          0         0         0

 RMAX          5.0
 *POLARIZATION    0.0      0.0      0.0

 POTENTIALS
   * ipot     Z          tag
       0        78        Pt
       1        78        Pt
       2        8          O


 ATOMS                               * this list contains 95 atoms
 *      x                 y                 z         ipot tag                 distance
      0.00000       0.00000       0.00000    0    Pt1                  0.00000
    -1.12095     -1.12095     -1.12095    2    O5.1                 1.94154
      1.12095       1.12095     -1.12095    2    O4.1                 1.94154
      1.12095     -1.12095       1.12095    2    O6.1                 1.94154
    -1.12095       1.12095       1.12095    2    O3.1                 1.94154
    -1.12095     -1.12095       3.36284    2    O5.2                 3.71776
    -1.12095       3.36284     -1.12095    2    O5.2                 3.71776
      3.36284     -1.12095     -1.12095    2    O5.2                 3.71776
      1.12095       1.12095       3.36284    2    O4.2                 3.71776
    -3.36284       1.12095     -1.12095    2    O4.2                 3.71776
      1.12095     -3.36284     -1.12095    2    O4.2                 3.71776
```

1.12095	3.36284	1.12095	2	O6.2	3.71776
-3.36284	-1.12095	1.12095	2	O6.2	3.71776
1.12095	-1.12095	-3.36284	2	O6.2	3.71776
3.36284	1.12095	1.12095	2	O3.2	3.71776
-1.12095	-3.36284	1.12095	2	O3.2	3.71776
-1.12095	1.12095	-3.36284	2	O3.2	3.71776
2.24189	2.24189	2.24189	1	Pt2.1	3.88307
-2.24189	2.24189	2.24189	1	Pt2.1	3.88307
2.24189	-2.24189	2.24189	1	Pt2.1	3.88307
-2.24189	-2.24189	2.24189	1	Pt2.1	3.88307
2.24189	2.24189	-2.24189	1	Pt2.1	3.88307
-2.24189	2.24189	-2.24189	1	Pt2.1	3.88307
2.24189	-2.24189	-2.24189	1	Pt2.1	3.88307
-2.24189	-2.24189	-2.24189	1	Pt2.1	3.88307
4.48378	0.00000	0.00000	1	Pt1.1	4.48378
-4.48378	0.00000	0.00000	1	Pt1.1	4.48378
0.00000	4.48378	0.00000	1	Pt1.1	4.48378
0.00000	-4.48378	0.00000	1	Pt1.1	4.48378
0.00000	0.00000	4.48378	1	Pt1.1	4.48378
0.00000	0.00000	-4.48378	1	Pt1.1	4.48378
-1.12095	3.36284	3.36284	2	O5.3	4.88609
3.36284	-1.12095	3.36284	2	O5.3	4.88609
3.36284	3.36284	-1.12095	2	O5.3	4.88609
-3.36284	1.12095	3.36284	2	O4.3	4.88609
1.12095	-3.36284	3.36284	2	O4.3	4.88609
-3.36284	-3.36284	-1.12095	2	O4.3	4.88609
-3.36284	3.36284	1.12095	2	O6.3	4.88609
1.12095	3.36284	-3.36284	2	O6.3	4.88609
-3.36284	-1.12095	-3.36284	2	O6.3	4.88609
3.36284	-3.36284	1.12095	2	O3.3	4.88609
3.36284	1.12095	-3.36284	2	O3.3	4.88609
-1.12095	-3.36284	-3.36284	2	O3.3	4.88609
3.36284	3.36284	3.36284	2	O5.4	5.82460
-5.60473	-1.12095	-1.12095	2	O5.4	5.82460
-1.12095	-5.60473	-1.12095	2	O5.4	5.82460
-1.12095	-1.12095	-5.60473	2	O5.4	5.82460
-3.36284	-3.36284	3.36284	2	O4.4	5.82460
5.60473	1.12095	-1.12095	2	O4.4	5.82460
1.12095	5.60473	-1.12095	2	O4.4	5.82460
1.12095	1.12095	-5.60473	2	O4.4	5.82460
5.60473	-1.12095	1.12095	2	O6.4	5.82460
1.12095	-5.60473	1.12095	2	O6.4	5.82460
1.12095	-1.12095	5.60473	2	O6.4	5.82460
-3.36284	3.36284	-3.36284	2	O6.4	5.82460

-5.60473	1.12095	1.12095	2	O3.4	5.82460
-1.12095	5.60473	1.12095	2	O3.4	5.82460
-1.12095	1.12095	5.60473	2	O3.4	5.82460
3.36284	-3.36284	-3.36284	2	O3.4	5.82460
4.48378	4.48378	0.00000	1	Pt1.2	6.34103
-4.48378	4.48378	0.00000	1	Pt1.2	6.34103
4.48378	-4.48378	0.00000	1	Pt1.2	6.34103
-4.48378	-4.48378	0.00000	1	Pt1.2	6.34103
4.48378	0.00000	4.48378	1	Pt1.2	6.34103
-4.48378	0.00000	4.48378	1	Pt1.2	6.34103
0.00000	4.48378	4.48378	1	Pt1.2	6.34103
0.00000	-4.48378	4.48378	1	Pt1.2	6.34103
4.48378	0.00000	-4.48378	1	Pt1.2	6.34103
-4.48378	0.00000	-4.48378	1	Pt1.2	6.34103
0.00000	4.48378	-4.48378	1	Pt1.2	6.34103
0.00000	-4.48378	-4.48378	1	Pt1.2	6.34103
-5.60473	-1.12095	3.36284	2	O5.5	6.63160
-1.12095	-5.60473	3.36284	2	O5.5	6.63160
-5.60473	3.36284	-1.12095	2	O5.5	6.63160
3.36284	-5.60473	-1.12095	2	O5.5	6.63160
-1.12095	3.36284	-5.60473	2	O5.5	6.63160
3.36284	-1.12095	-5.60473	2	O5.5	6.63160
5.60473	1.12095	3.36284	2	O4.5	6.63160
1.12095	5.60473	3.36284	2	O4.5	6.63160
-3.36284	5.60473	-1.12095	2	O4.5	6.63160
5.60473	-3.36284	-1.12095	2	O4.5	6.63160
-3.36284	1.12095	-5.60473	2	O4.5	6.63160
1.12095	-3.36284	-5.60473	2	O4.5	6.63160
5.60473	3.36284	1.12095	2	O6.5	6.63160
-3.36284	-5.60473	1.12095	2	O6.5	6.63160
1.12095	3.36284	5.60473	2	O6.5	6.63160
-3.36284	-1.12095	5.60473	2	O6.5	6.63160
5.60473	-1.12095	-3.36284	2	O6.5	6.63160
1.12095	-5.60473	-3.36284	2	O6.5	6.63160
3.36284	5.60473	1.12095	2	O3.5	6.63160
-5.60473	-3.36284	1.12095	2	O3.5	6.63160
3.36284	1.12095	5.60473	2	O3.5	6.63160
-1.12095	-3.36284	5.60473	2	O3.5	6.63160
-5.60473	1.12095	-3.36284	2	O3.5	6.63160
-1.12095	5.60473	-3.36284	2	O3.5	6.63160

END

Supplementary Data 2. Feff input file to generate Pt-Co path in Supplementary Figure 3.

It is generated using the crystal structure file of fcc Pt.

```
* This feff6 file was generated by Demeter 0.9.25
 * Demeter written by and copyright (c) Bruce Ravel, 2006-2016

 * --*--*--*--*--*--*--*--*--*--*--*--*--*--*--*--*--*--*--*--*--*--*--*--*--
 *    total mu*x=1:    2.420 microns,    unit edge step:    4.116 microns
 *    specific gravity:    21.483
 * --*--*--*--*--*--*--*--*--*--*--*--*--*--*--*--*--*--*--*--*--*--*--*--*--
 *    normalization correction:        0.00043 ang^2
 * --*--*--*--*--*--*--*--*--*--*--*--*--*--*--*--*--*--*--*--*--*--*--*--*--

 TITLE Mikiko Ochi
 TITLE    B-Site Deficiencies in A-site-Ordered Perovskite LaCu3Pt3.75O12

 HOLE           4      1.0      * FYI: (Pt L3 edge @ 11564 eV, second number is S0^2)
 *                 mphase,mpath,mfeff,mchi
 CONTROL     1          1         1         1
 PRINT        1          0         0         0

 RMAX          5.0
 *POLARIZATION    0.0      0.0      0.0

 POTENTIALS
   * ipot     Z          tag
       0        78        Pt
       1        78        Pt


 ATOMS                              * this list contains 55 atoms
 *      x                y                z          ipot tag                   distance
      0.00000       0.00000       0.00000    0    Pt2                   0.00000
      1.96090       1.96090       0.00000    1    Pt2.1                 2.77313
    -1.96090       1.96090       0.00000    1    Pt2.1                 2.77313
      1.96090     -1.96090       0.00000    1    Pt2.1                 2.77313
    -1.96090     -1.96090       0.00000    1    Pt2.1                 2.77313
      1.96090       0.00000       1.96090    1    Pt2.1                 2.77313
    -1.96090       0.00000       1.96090    1    Pt2.1                 2.77313
      0.00000       1.96090       1.96090    1    Pt2.1                 2.77313
      0.00000     -1.96090       1.96090    1    Pt2.1                 2.77313
      1.96090       0.00000     -1.96090    1    Pt2.1                 2.77313
    -1.96090       0.00000     -1.96090    1    Pt2.1                 2.77313
      0.00000       1.96090     -1.96090    1    Pt2.1                 2.77313
```

```
  0.00000   -1.96090   -1.96090   1   Pt2.1   2.77313
  3.92180    0.00000    0.00000   1   Pt2.2   3.92180
 -3.92180    0.00000    0.00000   1   Pt2.2   3.92180
  0.00000    3.92180    0.00000   1   Pt2.2   3.92180
  0.00000   -3.92180    0.00000   1   Pt2.2   3.92180
  0.00000    0.00000    3.92180   1   Pt2.2   3.92180
  0.00000    0.00000   -3.92180   1   Pt2.2   3.92180
  3.92180    1.96090    1.96090   1   Pt2.3   4.80320
 -3.92180    1.96090    1.96090   1   Pt2.3   4.80320
  1.96090    3.92180    1.96090   1   Pt2.3   4.80320
 -1.96090    3.92180    1.96090   1   Pt2.3   4.80320
  3.92180   -1.96090    1.96090   1   Pt2.3   4.80320
 -3.92180   -1.96090    1.96090   1   Pt2.3   4.80320
  1.96090   -3.92180    1.96090   1   Pt2.3   4.80320
 -1.96090   -3.92180    1.96090   1   Pt2.3   4.80320
  1.96090    1.96090    3.92180   1   Pt2.3   4.80320
 -1.96090    1.96090    3.92180   1   Pt2.3   4.80320
  1.96090   -1.96090    3.92180   1   Pt2.3   4.80320
 -1.96090   -1.96090    3.92180   1   Pt2.3   4.80320
  3.92180    1.96090   -1.96090   1   Pt2.3   4.80320
 -3.92180    1.96090   -1.96090   1   Pt2.3   4.80320
  1.96090    3.92180   -1.96090   1   Pt2.3   4.80320
 -1.96090    3.92180   -1.96090   1   Pt2.3   4.80320
  3.92180   -1.96090   -1.96090   1   Pt2.3   4.80320
 -3.92180   -1.96090   -1.96090   1   Pt2.3   4.80320
  1.96090   -3.92180   -1.96090   1   Pt2.3   4.80320
 -1.96090   -3.92180   -1.96090   1   Pt2.3   4.80320
  1.96090    1.96090   -3.92180   1   Pt2.3   4.80320
 -1.96090    1.96090   -3.92180   1   Pt2.3   4.80320
  1.96090   -1.96090   -3.92180   1   Pt2.3   4.80320
 -1.96090   -1.96090   -3.92180   1   Pt2.3   4.80320
  3.92180    3.92180    0.00000   1   Pt2.4   5.54626
 -3.92180    3.92180    0.00000   1   Pt2.4   5.54626
  3.92180   -3.92180    0.00000   1   Pt2.4   5.54626
 -3.92180   -3.92180    0.00000   1   Pt2.4   5.54626
  3.92180    0.00000    3.92180   1   Pt2.4   5.54626
 -3.92180    0.00000    3.92180   1   Pt2.4   5.54626
  0.00000    3.92180    3.92180   1   Pt2.4   5.54626
  0.00000   -3.92180    3.92180   1   Pt2.4   5.54626
  3.92180    0.00000   -3.92180   1   Pt2.4   5.54626
 -3.92180    0.00000   -3.92180   1   Pt2.4   5.54626
  0.00000    3.92180   -3.92180   1   Pt2.4   5.54626
  0.00000   -3.92180   -3.92180   1   Pt2.4   5.54626
END
```

Supplementary Data 3. Feff input file to generate Pt-Co path in Supplementary Figure 3.

It is generated using the crystal structure file of fcc Pt with 3 Pt atoms replaced by Co.

```
* This feff6 file was generated by Demeter 0.9.25
 * Demeter written by and copyright (c) Bruce Ravel, 2006-2016

 * --*--*--*--*--*--*--*--*--*--*--*--*--*--*--*--*--*--*--*--*--*--*--*--*--
 *    total mu*x=1:    6.110 microns,    unit edge step:    16.855 microns
 *    specific gravity:    10.238
 * --*--*--*--*--*--*--*--*--*--*--*--*--*--*--*--*--*--*--*--*--*--*--*--*--
 *    normalization correction:        0.00043 ang^2
 * --*--*--*--*--*--*--*--*--*--*--*--*--*--*--*--*--*--*--*--*--*--*--*--*--


 HOLE        4     1.0     * FYI: (Pt L3 edge @ 11564 eV, second number is S0^2)
 *              mphase,mpath,mfeff,mchi
 CONTROL     1      1      1      1
 PRINT       1      0      0      0

 RMAX        5.0
 *POLARIZATION   0.0     0.0     0.0

 POTENTIALS
   * ipot    Z       tag
      0      78      Pt
      1      27      Co
      2      78      Pt


 ATOMS                          * this list contains 55 atoms
 *     x            y            z        ipot tag              distance
     0.00000      0.00000      0.00000    0    Pt               0.00000
     1.96090      0.00000      1.96090    1    Co.1             2.77313
    -1.96090      0.00000      1.96090    1    Co.1             2.77313
     1.96090      0.00000     -1.96090    1    Co.1             2.77313
    -1.96090      0.00000     -1.96090    1    Co.1             2.77313
     1.96090      1.96090      0.00000    1    Co.1             2.77313
    -1.96090      1.96090      0.00000    1    Co.1             2.77313
     1.96090     -1.96090      0.00000    1    Co.1             2.77313
    -1.96090     -1.96090      0.00000    1    Co.1             2.77313
     0.00000      1.96090      1.96090    1    Co.1             2.77313
     0.00000     -1.96090      1.96090    1    Co.1             2.77313
     0.00000      1.96090     -1.96090    1    Co.1             2.77313
     0.00000     -1.96090     -1.96090    1    Co.1             2.77313
```

3.92180	0.00000	0.00000	2	Pt.1	3.92180
-3.92180	0.00000	0.00000	2	Pt.1	3.92180
0.00000	3.92180	0.00000	2	Pt.1	3.92180
0.00000	-3.92180	0.00000	2	Pt.1	3.92180
0.00000	0.00000	3.92180	2	Pt.1	3.92180
0.00000	0.00000	-3.92180	2	Pt.1	3.92180
1.96090	3.92180	1.96090	1	Co.2	4.80320
-1.96090	3.92180	1.96090	1	Co.2	4.80320
1.96090	-3.92180	1.96090	1	Co.2	4.80320
-1.96090	-3.92180	1.96090	1	Co.2	4.80320
1.96090	3.92180	-1.96090	1	Co.2	4.80320
-1.96090	3.92180	-1.96090	1	Co.2	4.80320
1.96090	-3.92180	-1.96090	1	Co.2	4.80320
-1.96090	-3.92180	-1.96090	1	Co.2	4.80320
1.96090	1.96090	3.92180	1	Co.2	4.80320
-1.96090	1.96090	3.92180	1	Co.2	4.80320
1.96090	-1.96090	3.92180	1	Co.2	4.80320
-1.96090	-1.96090	3.92180	1	Co.2	4.80320
1.96090	1.96090	-3.92180	1	Co.2	4.80320
-1.96090	1.96090	-3.92180	1	Co.2	4.80320
1.96090	-1.96090	-3.92180	1	Co.2	4.80320
-1.96090	-1.96090	-3.92180	1	Co.2	4.80320
3.92180	1.96090	1.96090	1	Co.2	4.80320
-3.92180	1.96090	1.96090	1	Co.2	4.80320
3.92180	-1.96090	1.96090	1	Co.2	4.80320
-3.92180	-1.96090	1.96090	1	Co.2	4.80320
3.92180	1.96090	-1.96090	1	Co.2	4.80320
-3.92180	1.96090	-1.96090	1	Co.2	4.80320
3.92180	-1.96090	-1.96090	1	Co.2	4.80320
-3.92180	-1.96090	-1.96090	1	Co.2	4.80320
3.92180	3.92180	0.00000	2	Pt.2	5.54626
-3.92180	3.92180	0.00000	2	Pt.2	5.54626
3.92180	-3.92180	0.00000	2	Pt.2	5.54626
-3.92180	-3.92180	0.00000	2	Pt.2	5.54626
3.92180	0.00000	3.92180	2	Pt.2	5.54626
-3.92180	0.00000	3.92180	2	Pt.2	5.54626
0.00000	3.92180	3.92180	2	Pt.2	5.54626
0.00000	-3.92180	3.92180	2	Pt.2	5.54626
3.92180	0.00000	-3.92180	2	Pt.2	5.54626
-3.92180	0.00000	-3.92180	2	Pt.2	5.54626
0.00000	3.92180	-3.92180	2	Pt.2	5.54626
0.00000	-3.92180	-3.92180	2	Pt.2	5.54626

END

Supplementary Data 4. The mass spectrometric results of various products in Table 2.

Entry 1

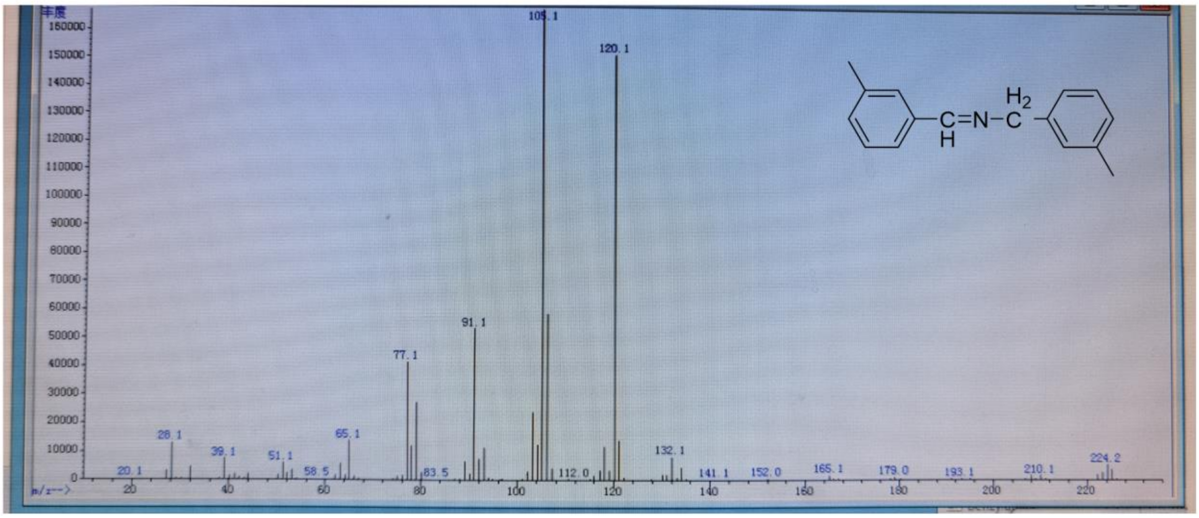

Entry 2

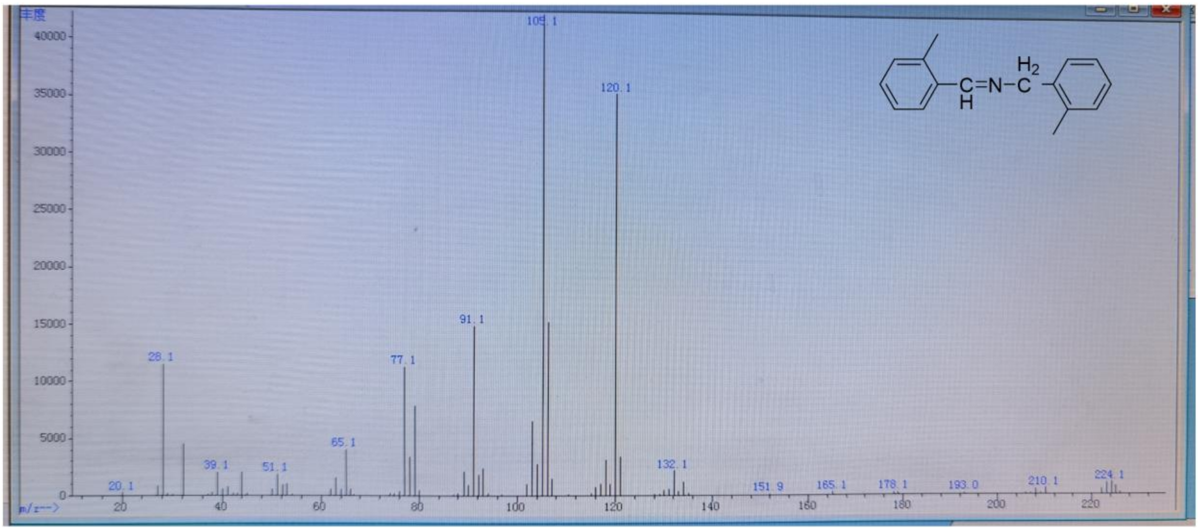

Entry 3

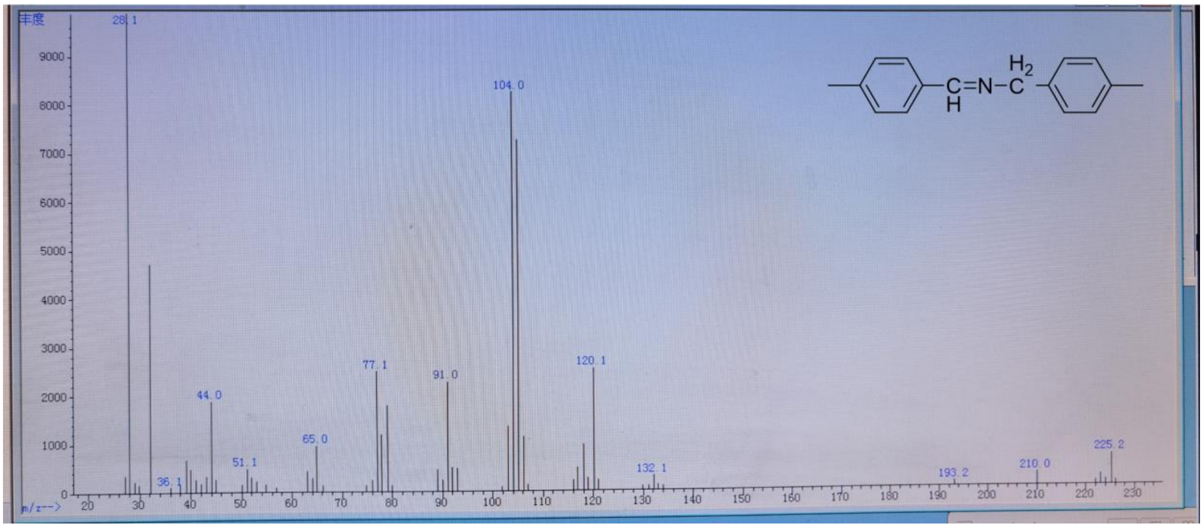

Entry 4

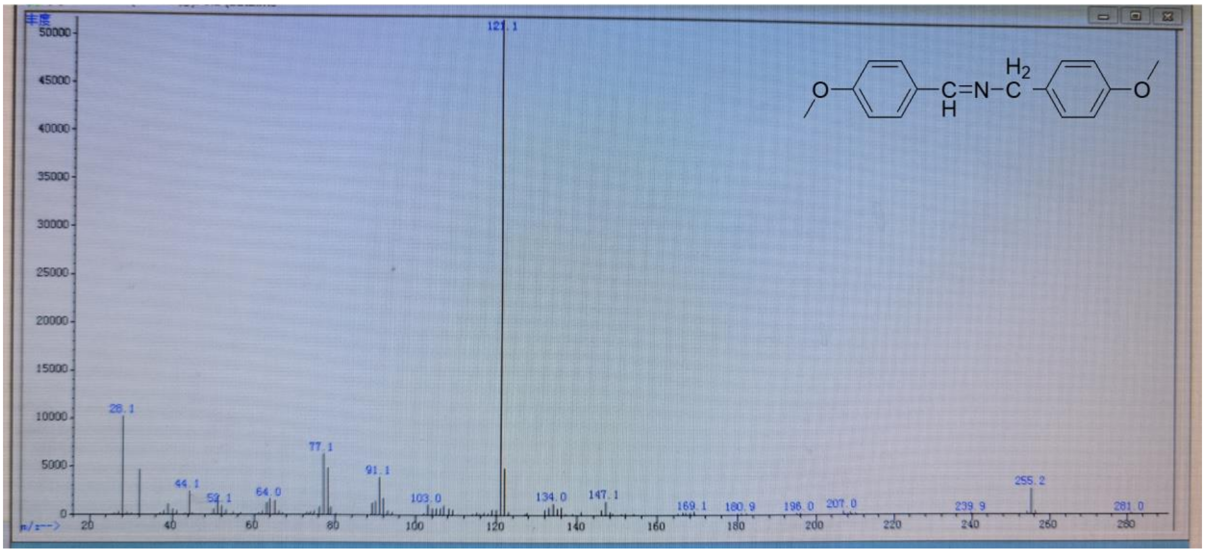

Entry 5

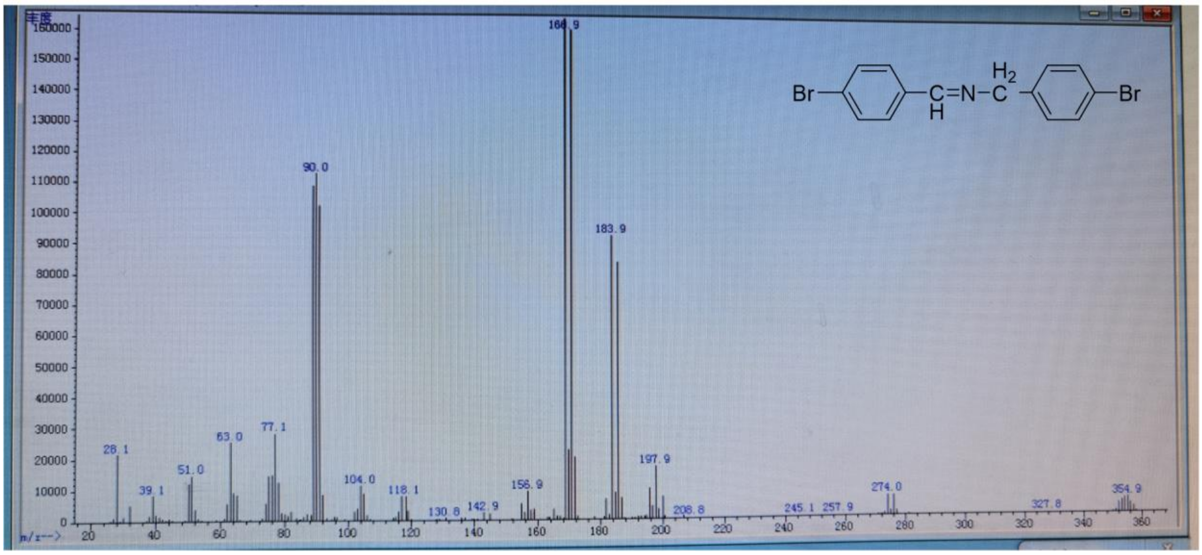

Entry 6

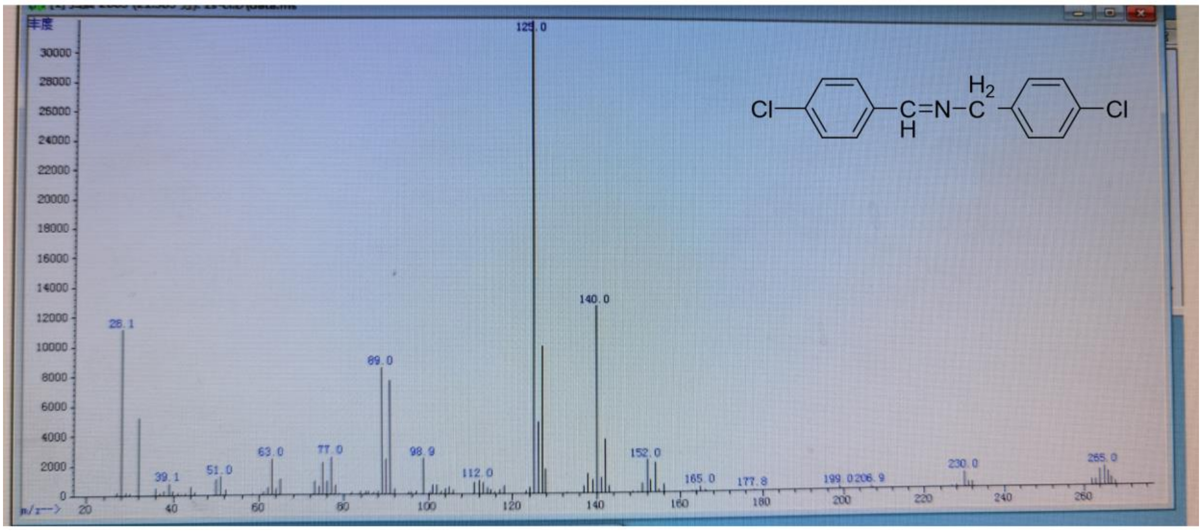

Supplement: Supplementary file 1 — Supplementary Information [file 41467_2021_23705_MOESM1_ESM.pdf]
